# How Photoactivation Triggers Protochlorophyllide Reduction: Computational Evidence of a Stepwise Hydride Transfer during Chlorophyll Biosynthesis

**DOI:** 10.1021/acscatal.2c00866

**Published:** 2022-03-21

**Authors:** Linus O. Johannissen, Aoife Taylor, Samantha J.O. Hardman, Derren J. Heyes, Nigel S. Scrutton, Sam Hay

**Affiliations:** Manchester Institute of Biotechnology and Department of Chemistry, The University of Manchester, Manchester M1 7DN, U.K.

**Keywords:** protochlorophyllide reductase (POR), time-dependent density functional theory (TD-DFT), enzyme mechanism, photocatalysis, hydride transfer, nicotinamide adenine dinucleotide phosphate (NADPH)

## Abstract

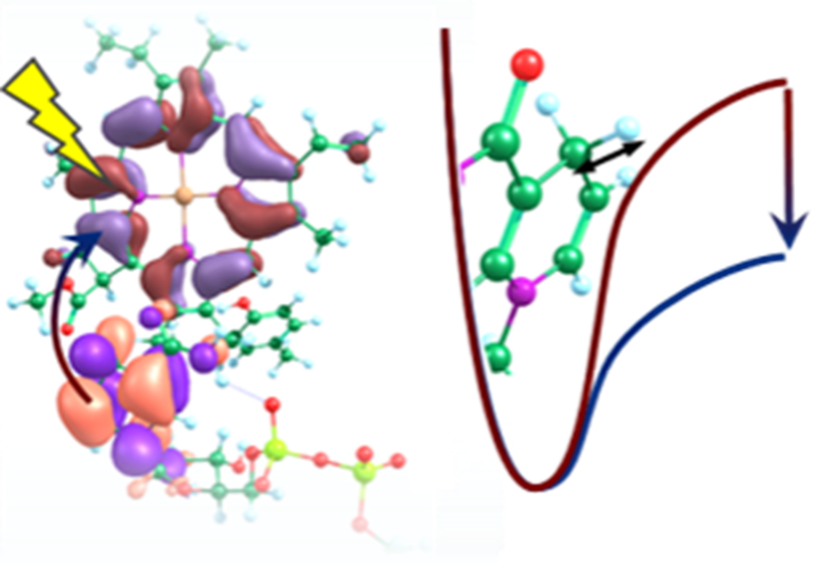

The photochemical reaction catalyzed by enzyme protochlorophyllide oxidoreductase (POR), a rare example of a photoactivated enzyme, is a crucial step during chlorophyll biosynthesis and involves the fastest known biological hydride transfer. Structures of the enzyme with bound substrate protochlorophyllide (PChlide) and coenzyme nicotinamide adenine dinucleotide phosphate (NADPH) have recently been published, opening up the possibility of using computational approaches to provide a comprehensive understanding of the excited state chemistry. Herein, we propose a complete mechanism for the photochemistry between PChlide and NADPH based on density functional theory (DFT) and time-dependent DFT calculations that is consistent with recent experimental data. In this multi-step mechanism, photoexcitation of PChlide leads to electron transfer from NADPH to PChlide, which in turn facilitates hydrogen atom transfer by weakening the breaking C–H bond. This work rationalizes how photoexcitation facilitates hydride transfer in POR and has more general implications for biological hydride transfer reactions.

## 1 Introduction

**Figure 1:**
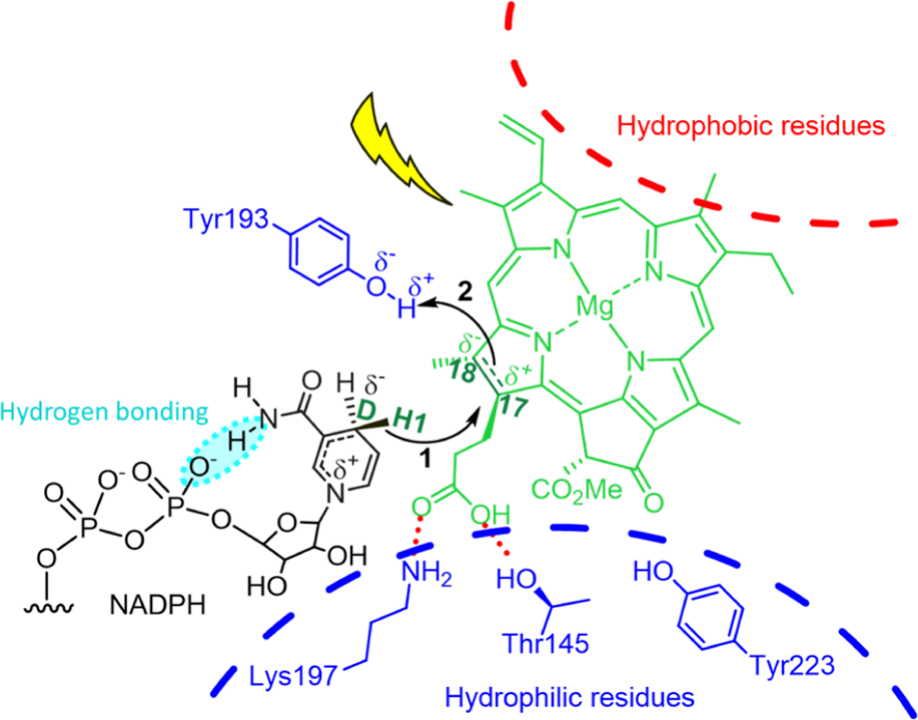
Schematic of the POR active site and the proposed reaction mechanism, with key atoms labeled: (1) hydride (H1) transfer from C_D_ to C17 and (2) PT from the proposed proton donor Tyr193 to C18.

Enzyme protochlorophyllide oxidoreductase (POR) catalyzes a vital light-dependent step during chlorophyll biosynthesis and acts as the trigger for plant germination.^1−5^ This is a rare example of an enzyme-catalyzed photochemical reaction, which has been extensively characterized through time-resolved spectroscopic techniques and single-turnover kinetic measurements.^6−10^ The reaction involves the reduction of a C=C double bond in substrate protochlorophyllide (PChlide), which proceeds in a stepwise manner via a hydride transfer (HYT) (a proton and two electrons) from coenzyme nicotinamide adenine dinucleotide phosphate (NADPH), followed by protonation of the resulting PChlide^–^ by an active site residue, likely Tyr193, or possibly a bound water molecule (Figure 1).^11^ The first step is triggered by the photoactivation of PChlide, which has been proposed to facilitate H-transfer by polarizing the C17=C18 double bond.^8^ The recently solved crystal structure of POR from *Thermosynechococcus elongatus* (TePOR) and our experimentally validated model of the ternary enzyme–substrate complex have suggested specific roles for a number of active site residues.^12^ For example, PChlide binding is aided by complementarity between hydrophobic and hydrophilic regions of the active site and the corresponding parts of PChlide, and this hydrophilic region, which also includes a number of water molecules, may play a role in stabilizing the dipole that has been proposed to arise across the C17=C18 bond after photoexcitation^8^ (Figure 1). This structural context now provides the opportunity to use computational approaches for a deeper understanding of POR catalysis, but it is essential to first understand the precise mechanism of the photochemistry and HYT chemistry between PChlide and NADPH. To this end, we have used density functional theory (DFT) and time-dependent DFT (TDDFT) calculations to derive a mechanism that is consistent with recent experimental data and fundamentally explains how photoexcitation leads to HYT.

It has recently been proposed that the POR-catalyzed HYT reaction, which is to the best of our knowledge the fastest reported enzymatic HYT, occurs as a stepwise process.^9^ Stepwise HYTs have been demonstrated in abiotic catalysis and biomimetic chemistry with NADH analogues,^13,14^ but this is the first known example of a biological stepwise HYT. A two-step, eT–PCET mechanism was suggested [electron transfer (eT) followed by a proton-coupled electron transfer (PCET)], although the precise nature of each step could not be determined, and the data were also consistent with a three-step eT–PT–eT mechanism (PT = proton transfer). It is also not clear whether these steps occur in the singlet or triplet excited state; the short lifetime of the first step suggests eT in the singlet state, but a reaction in the triplet state could explain why the quantum yield for the overall reaction^15^ is very similar to the ∼20–30% quantum yield of PChlide triplet formation after photoexcitation in solution.^7,16,17^

## 2 Computational Methods

### 2.1 DFT Model

Calculations were carried out on a model based on the published ternary structure of *T. elongatus* POR (TePOR),^12^ which includes PChlide, a truncated NADPH (“NDH”), and Tyr193 truncated at the Cβ, for a total of 137 atoms (Supporting Information Figure S1 shows the energy minimized model in the S0 state). NADPH was truncated after the diphosphate group, which is hydrogen bonded to the amide of the nicotinamide moiety in both the crystal structure and during molecular dynamics (MD) simulations of the POR ternary complex model (Figure 1).^12^ Four atoms were kept fixed during all calculations, indicated with “*” in Supporting Information Figure S1. Prior to calculations, Tyr193 was rotated around the Cα-Cβ bond to position the hydroxyl group toward C18 ready for protonation, preventing too much movement of PChlide without fixing additional atoms in PChlide. Tyr193 exhibits a wide range of conformations during MD simulations, performed using the methods and parameters described in our previous work^12^ (Supporting Information Figure S2). Such a conformational change is likely required prior to protonation, yet it is not clear at what point during the reaction this occurs. Note, however, that protonation might involve bridging water molecule(s), and other proton donors have also been proposed based on alternative PChlide binding poses,^18,19^ although Tyr193 is the most likely proton donor in our published model.^12^ All calculations were performed in Gaussian^16^ 16 revision A03.^20^ The M06-2X hybrid functional,^21^ which has been shown to perform well for charge transfer calculations^22^ and for triplet energies and singlet-to-triplet transitions,^23−25^ was employed for all energy minimizations, with the 6-31G(d,p) basis sets on all atoms. Single-point calculations were then performed using a range of functionals as well as the TZVP basis set in order to identify the best method for modeling eT in this model system. Polarizable continuum solvation was used to model water solvation (ε = 80) as the polar groups of PChlide and NADPH are surrounded by a large number of water molecules and polar residues in our published structure.^12^

To model eT and the subsequent H-transfer from oxidized NADPH to reduced PChlide, we generated “pre-eT” and “post-eT” geometries. Energy minimization of the ground (S0) state using a spin multiplicity of 1 produced a structure corresponding to a pre-eT state (Supporting Information Table S1). For the post-eT state, the system was energy-minimized as a triplet with a spin multiplicity of 3, with orbital swapping of the β highest occupied molecular orbital (HOMO) and lowest unoccupied molecular orbital (LUMO) orbitals: without orbital swapping, both singly occupied molecular orbitals (SOMOs) were located on PChlide, but after orbital swapping, one SOMO was localized on NDH and the other on PChlide. This reproduces the main structural changes that occur after one-electron reduction of a PChlide model and one-electron oxidation of an NADPH model (Supporting Information Table S2) optimized at the same level of theory and similar PChlide charges (Supporting Information Table S1). TDDFT was then used for excited state calculations on both the pre- and post-eT structures, solving for the lowest 10 states. Excited-state energies were computed using a range of functionals, with M06 most closely reproducing the eT energy obtained from calculations on isolated PChlide/PChlide^–^ and NADPH/NADPH^+^ molecules (Table S3) and hence used for further TDDFT calculations.

The outer (solvent) reorganization energies for eT were calculated from the difference between the nonequilibrium and equilibrium solvation energies for the appropriate electronic states. The nonequilibrium solvation was calculated by using the inertial charges for the lower-energy electronic state and performing a state-specific solvent response correction^26^ on the higher electronic state. The inner reorganization energies were calculated from the difference in energies between the pre- and post-eT geometries in the (NDH^+^/PChlide^–^) electronic state for reorganization during eT and the S0 (NDH/PChlide) state for reorganization during back-eT.

To model H-transfer, relaxed potential energy scans were initially performed with the M06-2X functional, using a reaction coordinate, *z*, defined as the difference between the breaking and forming bonds. For H-transfer after eT [in the (NDH^+^/PChlide^–^) state] in the post-eT geometry, a scan was performed starting from the post-eT structure, again using a spin multiplicity of 3. Barrier heights were then calculated first with single-point TDDFT calculations on intermediate structures obtained from these scans, and then with TDDFT optimization of certain points along this barrier using the functional that performed the best for the eT energy in this system (M06; see above), as well as energy minimization of the S0 state to derive relative energies (Supporting Information Figure S3). Zero-point energy corrections and enthalpic and free energy corrections were derived from normal mode calculations on these structures in the appropriate electronic state. Note that since TDDFT energy minimization is very slow, loose convergence criteria were used (energy minimization plots are shown in Supporting Information Figure S3B), which means that these thermal corrections are not precise. However, the aim of this study is not the accurate reproduction of reaction barriers, and these calculations are adequate for a semi-quantitative mechanistic study.

### 2.2 Quantum Mechanics/Molecular Mechanics Model

A model was constructed from the same starting structure as that of the DFT model, composed of the entire enzyme and water molecules whose oxygen atom is within 20 Å of PChlide or NADPH, for a total of 11 040 atoms (Supporting Information Figure S5A). The majority of the molecular mechanics (MM) region was kept fixed during energy minimizations, except for residues with at least one atom within 10 Å and water molecules whose oxygen atom is within 8 Å of PChlide or NADPH, for a total of 2787 unrestrained atoms (Supporting Information Figure S5B). The quantum mechanics (QM) region was composed of PChlide, the two water molecules directly ligated to the PChlide Mg^2+^, and NADPH, which was truncated so as to include the diphosphate group (Supporting Information Figure S5C), with a link atom between the MM and QM atoms of NADPH. Calculations were performed using the ONIOM method^27,28^ with electronic embedding at the same level of theory for the QM atoms as that for the DFT model and Amber parameters^29^ for the MM region. Force constants were calculated at the start of each optimization (using opt = calcfc) to prevent drastic changes to the structure of the solvent along the reaction coordinate, which meant that TDDFT minimization or normal mode calculations on the excited states were not feasible. For each excited sate, Mulliken charges were used to determine whether an eT had occurred to PChlide.

### 2.3 Bond Dissociation Energies

Bond dissociation energies (BDEs) and reaction energies for different possible HYT mechanisms were calculated for isolated PChlide and NADPH molecules. Energy minimization was performed at the M06-2X/6-31G(d,p)/PCM(ε = 80) level of theory, with single-point energies calculated at the M06-2X/6-311+G(d,p)/PCM(ε = 80) level of theory, accounting for basis set superposition error using the counterpoise method.^30,31^

## 3 Results and Discussion

### 3.1 Benchmarking

To select a functional most suitable to study this system, we first benchmarked the TDDFT calculations against DFT calculations on eT between isolated PChlide/PChlide^–^ and NADPH/NADPH^+^ molecules. In our TDDFT calculations, eT from NDH to PChlide occurs in most cases in excited states above the lowest singlet excited state, S1, and the precise energy level where eT occurs depends on the geometry and the functional used (Supporting Information Table S3). We will use (NDH/PChlide), (NDH/PChlide)*, and (NDH^+^/PChlide^–^) to denote the ground (S0) state, the excited state without eT from NDH to PChlide, and the electronic state after eT from NDH to PChlide, respectively; (NDH^+^/PChlide^–^) is defined as the excited state dominated by transfer from a molecular orbital centered on NDH to an orbital centered on PChlide. A range of functionals and different basis sets (Supporting Information Table S3) produce very similar energies for the singlet (NDH/PChlide)*, consistent with previous calculations on PChlide.^11,32^ There is, however, a large variation in the triplet (NDH/PChlide)* and (NDH^+^/PChlide^–^) energies, although this does not affect the general trends. Since the (NDH^+^/PChlide^–^) energy obtained with M06 in the post-eT geometry is very similar to the ∼180 kJ mol^–1^ obtained for eT from NADPH to PChlide using a range of DFT methods (Supporting Information Table S4), this will be used in further discussion.

### 3.2 Concerted or Stepwise HYT?

**Figure 2:**
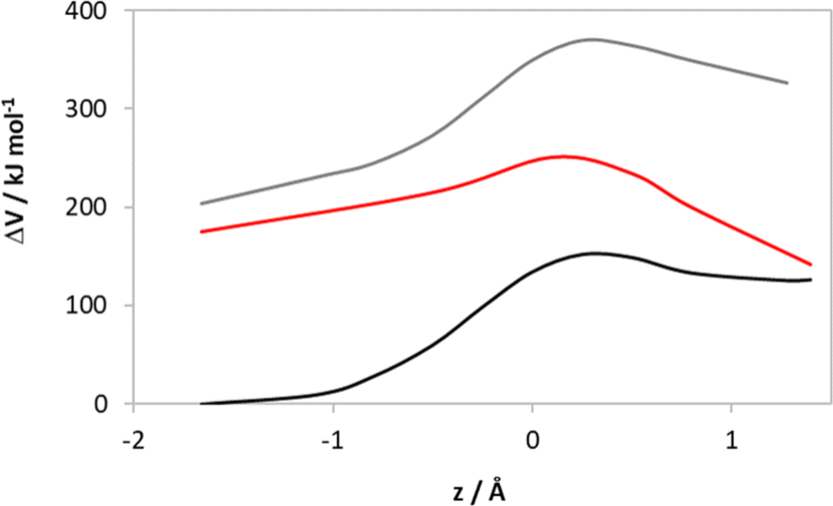
Potential energy barriers for concerted HYT in the ground (black) and (NDH/PChlide)* states (gray) in the pre-eT geometry and (NDH^+^/PChlide^–^) state (red) after TDDFT energy minimization. See also, Supporting Information Figure S3.

H-transfer scans and TDDFT calculations suggest that the barrier for a concerted HYT from the excited state is prohibitively large (Figure 2 and Supporting Information Figure S3). For ground-state HYT, the potential energy barrier is Δ*V*^‡^ = 151 kJ mol^–1^, and for (NDH/PChlide)*, Δ*V*^‡^ = 166 kJ mol^–1^. However, on the (NDH^+^/PChlide^–^) electronic surface, the barrier decreases to Δ*V*^‡^ = 54 kJ mol^–1^. In this, geometry conversion from (NDH/PChlide)* to (NDH^+^/PChlide^–^) by eT is uphill by 20 kJ mol^–1^, but even taking this into account, a stepwise mechanism is more likely than a HYT (Supporting Information Figure S3). However, these scans were performed using the pre-eT geometry, and eT will be accompanied by a reorganization to the post-eT geometry, as discussed in the next section. Note that since (NDH^+^/PChlide^–^) is not the lowest energy electronic state for the system, this diradical species formed by eT is technically an excited state. However, since a formal charge transfer has taken place, NDH^+^ and PChlide^–^ are both individually in their electronic ground states, and the reorganization energy that accompanies eT and back-eT increases the kinetic stability of (NDH^+^/PChlide^–^) relative to (NDH/PChlide)*.

### 3.3 Step 1: Electron Transfer to PChlide

**Figure 3:**
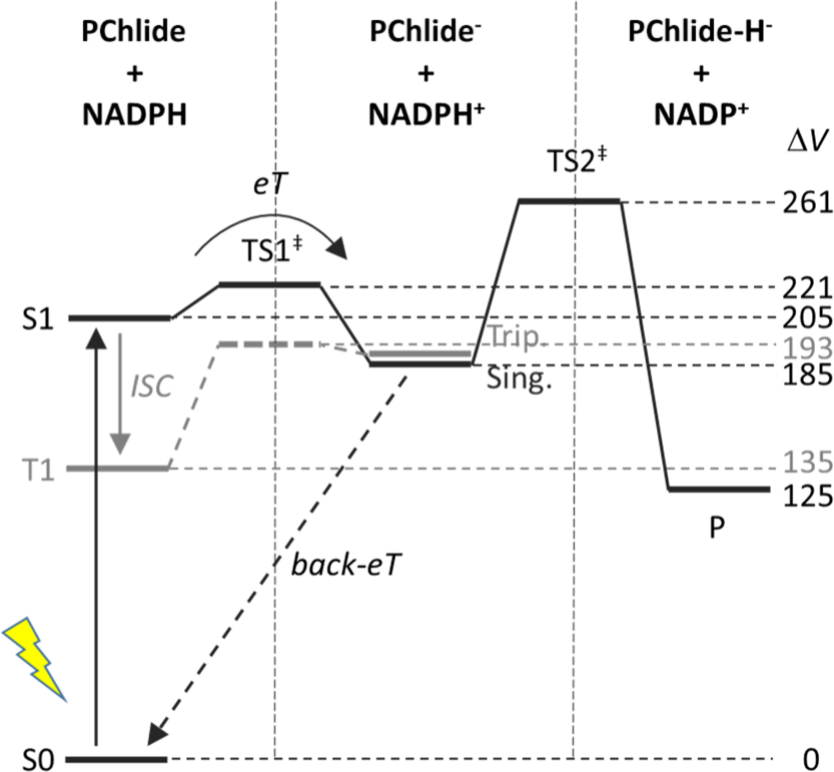
Reaction profile for proposed stepwise HYT, with potential energies in kilojoules per mole.

The energy of the singlet (NDH/PChlide)* state is calculated to be 205 kJ mol^–1^, and intersystem crossing to the triplet state decreases this to 135 kJ mol^–1^ (Figure 3). In this same geometry, the singlet and triplet (NDH^+^/PChlide^–^) energies are 225 and 224 kJ mol^–1^, respectively, but after reorganization to the post-eT geometry, the (NDH^+^/PChlide^–^) energies decrease to 185 and 188 kJ mol^–1^, respectively. Therefore, despite very similar energies for the singlet and triplet (NDH^+^/PChlide^–^) states, the low energy for triplet (NDH/PChlide)* means that eT in the triplet state is endothermic (+53.7 kJ mol^–1^), while eT in the singlet state is exothermic (−20.3 kJ mol^–1^), as illustrated in Figure 3. Together with a weak spin–orbit coupling of 1.13 cm^–1^ (calculated using PySOC;^33^Supporting Information Table S5), this suggests that eT is much more likely in the singlet state. Natural transition orbitals^34^ for this eT are shown in Supporting Information Figure S4.

The barrier for eT was calculated from the Marcus equation and the reorganization energy (Supporting Information Table S6). The total reorganization energy is λ = 94.7 kJ mol^–1^, resulting in potential energy barriers of Δ*V*^‡^ = 16.6 and 58.1 kJ mol^–1^ for singlet and triplet eTs, respectively. The kinetic isotope effect (KIE) was then calculated from the zero-point energy and thermally corrected barriers (Table 1). Note that these corrections are not precise since structures are not energy-minimized in the relevant excited states, but much of the error is cancelled when calculating KIEs, which are consistent with the experimentally observed KIE for the first phase of the mechanism after photoactivation (see Section 3.7).

**Table 1:** Barriers (Δ*E* in kJ mol^–1^) to eT and HAT and Their 1° KIEs Computed Using the Zero-Point Energy Corrected and Enthalpic and Gibbs Free Energies

	ZPE corrected	enthalpy	Gibbs free energy
1. eT			
Δ*E*(H)^a^	14.8	13.5	18.4
KIE^b^	1.07	1.08	1.07
2. HAT			
Δ*E*(H)^a^	64.0	64.1	66.2
KIE^b^	4.15	4.08	4.44

a For the H isotopologue of NDH.

b Deuterium KIEs computed with *S*-[^2^H]_4_-NDH.

### 3.4 Step 2: Proton Transfer or Proton-Coupled Electron Transfer?

After the initial eT, HYT could be completed by either a two-step PT–eT mechanism or a single PCET step. In either case, the second step involves transfer of the hydrogen nucleus, so this step was modeled using relaxed potential energy scans starting from the post-eT geometry. The barrier on the (NDH^+^/PChlide^–^) surface was then obtained from TDDFT energy-minimized structures from this scan (Figure 2 and Supporting Information Figure S3). This resulted in a barrier with Δ*V*^‡^ = 76.2 kJ mol^–1^ and Δ*H*^H‡^ = 64.1 kJ mol^–1^ (from normal mode thermal corrections). The computed KIEs are similar when using zero-point energy corrections or the computed enthalpy or free energies (Table 1) and agree well with experimental values (see Section F). Note that this barrier is somewhat larger than that for H-transfer on the (NDH^+^/PChlide^–^) surface in the pre-eT geometry (see Section 3.2) because the energy minimization of structures on the (NDH^+^/PChlide^–^) surface affects the transition state less than the reactant state but is still significantly lower than the barrier for ground- or excited-state HYT. The HYT product (ND^+^/PChlide-H^–^) lies on the ground-state surface so that a relaxation to this state is required to finalize the reaction. The energy gap (vertical excitation energy) is only 1.5 kJ mol^–1^, so an adiabatic transition to the ground-state product (ND^+^/PChlide-H^–^) can occur.

**Figure 4:**
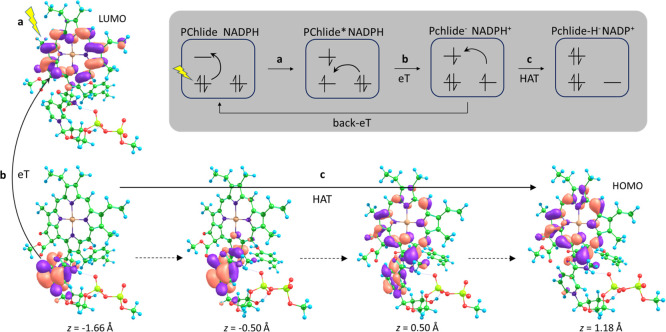
Mechanism for the proposed stepwise HYT from NADPH to PChlide: (a) photoexcitation of PChlide to the S1 singlet excited state; (b) eT from NDH to the LUMO on PChlide; and (c) HAT from NDH^+^ to PChlide^–^. As the system progresses along the reaction coordinate, *z* (dotted arrows), a second electron is transferred from the NDH SOMO to the PChlide SOMO, which becomes the HOMO in PChlide-H^–^. The natural transition orbitals (with contributions of >0.99) are shown for every structure apart from the ground-state product (*z* = 1.18 Å), for which the HOMO is shown. Inset: diagrammatic representation of the electronic rearrangements during steps a–c.

Inspection of the change in the molecular orbitals as the H nucleus is transferred along the reaction coordinate suggests that this is a PCET as the H-transfer is accompanied by a transfer of the electron density from NDH^+^ to PChlide^–^ (Figure 4c). During the initial eT step, the first electron is transferred from an orbital centered on NDH to one centered on PChlide; the second electron is then transferred during the H-transfer step from the now SOMO on NDH to the SOMO on PChlide. This molecular orbital is transferred along the σ-orbitals on the donor and acceptor carbon atoms and occurs gradually with the H-transfer, which is indicative of adiabatic PCET or hydrogen atom transfer (HAT).^35,36^

### 3.5 Back-Electron Transfer

Experimentally, the first step was determined to occur with a lifetime of ∼0.7 ns, with subsequent steps occurring over tens to hundreds of nanoseconds,^9^ which is qualitatively consistent with our computed barrier for the eT step being significantly lower than the barrier for the second step. However, in order for our proposed mechanism to be viable, the second step needs to outcompete back-eT (or since the reaction quantum yield is ∼30%,^15^ it must outcompete back-eT at least ∼30% of the time). After the formation of PChlide^–^, eT will return the system to the S0 state as eT from a lower orbital to reform the PChlide S1 state is highly unlikely (Figures 3 and 4, inset). The driving force for back-eT is −182 kJ mol^–1^, which is significantly larger than the reorganization energy λ = 99.1 kJ mol^–1^. This means that back-eT would occur in the Marcus inverted region where a smaller reorganization energy leads to an increased activation energy, which would reduce the rate of the back-eT reaction. In our simple model, the barrier for back-eT is very similar to that for eT (17.4 and 16.6 kJ mol^–1^, respectively), but the reorganization energies were calculated using a water solvation model. It is well-established that enzymes decrease the reorganization energy relative to the reference reaction in water, which would reduce the rate of back-eT.^37^ This strategy is employed by photosystem II to minimize unproductive charge recombination events during photosynthesis.^38^

### 3.6 QM/MM Calculations

In order to explore the effects of the protein environment on the photochemistry, QM/MM ONIOM^27,28^ calculations were performed on the POR ternary complex. Unfortunately, it was not possible to optimize a (NADPH^+^/PChlide^–^) geometry with the same orbital swapping technique as that for the DFT model: while the polarizable continuum solvent is recalculated in response to a change in the electron distribution, the MM region is fixed during the optimization of the molecular orbitals after the orbital swap. Additionally, TDDFT energy minimizations and frequency calculations were too computationally expensive, so our analysis is therefore limited to potential energies for the (NADPH/PChlide) geometry and the corresponding barrier and thus should be treated semi-quantitatively. Nevertheless, some interesting observations can be made by comparison to the results for the (NDP/PChlide) geometry of the DFT model (Table 2). The ground-state potential energy barrier is much higher for the QM/MM model than that for the DFT model. However, this is not unreasonable since the enzyme does not catalyze a ground-state HYT but primarily needs to stabilize the barrier for HAT as this is rate-limiting. Additionally, it is likely that the chosen MD structure is not in a reactive conformation, which might require additional reorganization. In the QM/MM model, the excitation energy is ∼20 kJ mol^–1^ lower than that in the DFT model. It is well-known from mutagenesis studies that residues in the POR active site can have significant effects on the excited state chemistry of PChlide,^5^ and the HAT barrier is sensitive to the inclusion of surrounding residues in the TDDFT calculation; for example, inclusion of Tyr193 or Phe233 decreases Δ*V*^‡^ by 5 kJ mol^–1^ and that of Phe247 by 11 kJ mol^–1^. The conformation of these residues will likely be important also, so the actual barrier is likely significantly lower. These residues increase the energy of the (NADPH^+^/PChlide^–^) intermediate, but this is reasonable as a stable intermediate is not desirable for an efficient mechanism. In the DFT model, the HAT barrier is 79 kJ mol^–1^ lower than the ground state barrier, while for the QM/MM model, it is lower by 88 kJ mol^–1^ when Tyr193 is included. There are likely important effects that our model does not properly describe, for example, polarization and hydrogen bonding effects that stabilize the charge separation, and it is possible that the polarizable continuum of the DFT model may better describe some of these effects. Further work will be required to properly determine the role of the protein environment on the photochemistry of POR.

**Table 2:** Energies (in kJ mol^−1^) for QM/MM and DFT Models in the Pre-eT Geometry

model	Δ*V*^‡^(S_0_)	S_1_	Δ*E*(eT)	Δ*V*^‡^(HAT)
DFT	133.6	204.9	19.8	53.7
QM/MM	171.3	183.2	24.0	88.0
+Y193^a^		183.0	28.8	83.0
+F233^a^		182.7	31.1	82.7
+F247^a^		182.7	36.5	77.2

a These residues were included in the QM region for the purpose of excited state TDDFT calculations.

### 3.7 Comparison to Experimental Data

In our model, HYT occurs in a two-step eT–PCET, or more specifically eT–HAT, mechanism. This agrees with the multi-step mechanism previously proposed based on time-resolved spectroscopy, which was consistent with either a two- or three-step mechanism:^9^ while three phases were observed, each phase does not necessarily correspond to a distinct mechanistic species due to the kinetic complexity of multistep processes. There was no KIE observed on the fastest phase, but KIEs of 1.4–1.9 were seen on the following two phases.^9^ The first KIE ∼1 agrees well with our computed KIE of 1.07 for the eT step, and our low computed barrier for eT agrees with this being by far the fastest step.^9^ The KIEs for the next phases are not directly comparable to our computed values. A breakpoint has previously been identified in the kinetic data for HYT in TePOR, with a nearly temperature-independent KIE of ∼5 below −27 °C, which then decreases at higher temperatures.^11,39^ This is believed to arise from dynamic donor–acceptor distance sampling that lowers the barrier at higher temperatures. Our calculations, which do not take such dynamics into account, produced a KIE of ∼4.1–4.4 (Table 2), which is similar to the measured KIE observed below the breakpoint.^11,39^ Note that the computed enthalpic barrier for HAT, Δ*H*^H‡^ = 64.1 kJ mol^–1^, is much higher than the experimental barrier for the overall HYT (Δ*H*^H‡^ = 27.2 kJ mol^–1^),^11,39^ although this is to be expected since factors such as the electrostatic effect of the enzyme were not included here and could not be properly accounted for in our QM/MM calculations.

An alternative mechanism for POR was proposed during the reviewing process for this manuscript, which involves eT from Tyr193 to the photoactivated PChlide.^19^ This mechanism was calculated to have a lower barrier (Δ*G*^‡^ = 46.4 kJ mol^–1^), but a redox active Tyr193 is not consistent with mutagenesis experiments, which have shown that while Tyr193 mutants show decreased activity at physiological temperatures, there is no significant difference in the rate of HYT in the Y193F variant at cryogenic temperatures compared to that in the wild type.^40^ Therefore, while it is possible that our calculations overestimate the barrier because a different mechanism is involved, we believe that this remains the most likely mechanism based on our previously published structure.^12^

Photoactivation of PChlide has been proposed to result in the formation of an internal charge transfer (ICT) state^6,7^ as electron density is redistributed from the HOMO localized on the inner porphyrin ring to the LUMO delocalized across the inner and outer porphyrin.^32^ It has been proposed that the ICT state converts to a “reactive ICT” species before H-transfer occurs^6,7^ and that this results in a weakened, polarized C17=C18 double bond that is more easily cleaved during H-transfer.^8^ This is supported by a downshift in the C=C double bond region of the infrared (IR) spectrum relative to the ground state.^8^ In order to test this hypothesis, we modeled different electronic states of an isolated PChlide molecule: the ground state (S0), lowest singlet excited state (S1), and single-electron reduced state (PChlide^–^) (Supporting Information Figure S6 and Supporting Information Table S1). S1 and PChlide^–^ have a significantly different charge distribution around the porphyrin compared to S0, consistent with the proposed internal charge transfer. However, the vibrational stretching frequency for the C17=C18 double bond is upshifted by 12–15 cm^–1^ in S1 compared to that in S0 (Supporting Information Table S7) and in PChlide^−^, suggesting that this bond is in fact strengthened by photoactivation as well as by eT. However, the calculations are qualitatively consistent with the observed ∼10 cm^–1^ downshift in the C=C region of the IR spectra for the “reactive ICT” state^8^ as the stretching frequencies of the majority of the computed C=C double bonds in the porphyrin ring decrease, and the average decrease is ∼15 cm^–1^. We also observe a decrease of ∼80 cm^–1^ for the carbonyl C=O group in PChlide^–^, consistent with the observed downshift in the carbonyl region of the IR spectra (Supporting Information Figure S7 and Table S7).^8^

Since these data suggest that H-transfer is not facilitated by weakening/polarization of the C17=C18 double bond, we analyzed other factors that may be involved by calculating the reaction energies and relevant BDEs for possible HYT mechanisms between NADPH and PChlide (Supporting Information Figure S6, Table 3). This suggests that for a mechanism initiated by a PCET (mechanism 2) or where PT follows initial eT (mechanism 3), the BDE of the H-donor increases significantly. On the other hand, oxidation of NADPH leads to a significant reduction in the BDE for the homolytic cleavage of the NADPH^•^ C4–H bond (mechanism 4). The corresponding BDE for PChlide-H^•^ (homolytic cleavage of the PChlide-H^•^ C17–H bond) increases somewhat, but this is more than offset by the decrease in the reaction energy due to the energetic cost of the initial eT. This suggests that photoactivation facilitates HYT by initiating eT from NADPH to PChlide, which has two effects: (i) the next step becomes exothermic and (ii) the BDE for homolytic cleavage of the breaking C–H bond decreases. Note that a photoexcited oxidant in a biomimetic reaction was also recently shown to work by decreasing the C–H BDE,^41^ so this appears to be an effective strategy for light-activated H-transfer reactions and should be considered when designing artificial light-harvesting systems; to this end, simple BDE calculations can be very informative. The fact that the POR reaction involves the fastest known biological HYT is likely due to the photochemical nature of this reaction since photoactivation triggers eT and hence the decrease in H-donor BDE. However, since photoactivation of PChlide does not change the chemical nature of NADPH, it seems likely that a stepwise mechanism is more general among the >400 enzyme-catalyzed reactions depending on cofactor NAD(P)H,^42^ although the precise mechanism of each reaction will depend on the redox properties of the acceptor molecule and the enzyme environment.

**Table 3:** BDEs and Reaction Energies (Δ*E*_step_^1^) in kJ mol^−1^ for Distinct Steps during Possible HYT Mechanisms

step	mechanism	BDE	Δ*E*_step_
1. HYT			
1a. HYT	NADPH → NADP^+^ + H^–^	200	102
	PChlide-H^–^ → PChlide + H^–^	98.4	
2. PCET–eT			
2a.HAT	NADPH → NADP^•^ + H^+^	330	142
	PChlide-H^•^ → PChlide	189	
2b. eT	PChlide-H^-^ → PChlide-H^•^		–43.7
	NADP^•^ → NADP^+^		
3. eT–PT–eT			
3a. eT	NADPH → NADPH^+^		183
	PChlide → PChlide^–^		
3b. PT	NADPH^+^ → NADP^•^+ H^+^	696	–41.4
	PChlide-H^•^ → PChlide^–^ + H^+^	731	
3c. eT	NADP^•^ → NADP^+^		–43.7
	PChlide-H^–^ → PChlide-H^•^		
4. eT–PCET			
4a. eT	NADPH → NADPH^+^		183
	PChlide → PChlide^–^		
4b.HAT	NADPH^+^ → NADP^+^+ H^•^	131	–85.0
	PChlide-H^–^ → PChlide^–^ + H^•^	216	

## Conclusions

In summary, we have described a complete mechanism for the stepwise HYT from NADPH to PChlide, which is qualitatively consistent with recent mechanistic data. We propose that HYT occurs in a stepwise manner from the photoexcited PChlide via eT from NADPH to PChlide in the singlet state, followed by an HAT from NADPH^+^ to PChlide^–^, and that unproductive back-eT is inhibited by the Marcus inverted region. This mechanism is consistent with the published spectroscopic data, and our calculations agree with the experimental interpretation that eT is the fastest step. In contrast to a previous interpretation, our calculations suggest that photoactivation does not weaken the C17=C18 bond that becomes reduced but instead that eT facilitates H-transfer by decreasing the BDE for homolytic C–H bond cleavage in oxidized NADPH. This explains how photoactivation triggers the fastest known biological HYT, by significantly reducing the BDE for H-transfer.

## Supporting Information Available

Additional methodological details, benchmarking, and computational analysis (PDF)
