# Supplementary material for: How Photoactivation Triggers Protochlorophyllide Reduction: Computational Evidence of a Stepwise Hydride Transfer during Chlorophyll Biosynthesis
Source: ACS Catal. 2022 Mar 21;12(7):4141–8. doi: 10.1021/acscatal.2c00866 (PMC9098174; doi:10.1021/acscatal.2c00866)
Supplement: Supplementary file 1 — cs2c00866_si_001.pdf [file cs2c00866_si_001.pdf]

Supporting Information

How Photoactivation Triggers Protochlorophyllide Reduction: Computational Evidence of a Stepwise Hydride Transfer During Chlorophyll Biosynthesis

Linus O. Johannissen,* Aoife Taylor, Samantha J.O. Hardman, Derren J. Heyes, Nigel S. Scrutton and Sam Hay*

Manchester Institute of Biotechnology and Department of Chemistry, The University of Manchester, Manchester M1 7DN, UK

*Linus O. Johannissen: linus.johannissen@manchester.ac.uk

*Sam Hay: sam.hay@manchester.ac.uk

Supplementary Figures

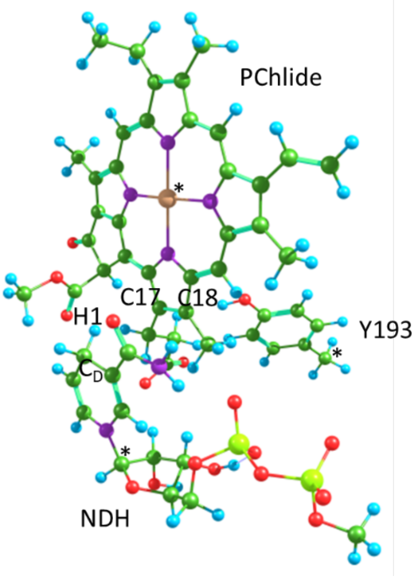

Figure S1. Model system used for barrier calculations and TDDFT calculations, energy minimised in the S0 state. * denotes atoms kept fixed during energy minimisations. CD is the hydride donor carbon atom, H1 the transferring hydride, C17 the hydride acceptor carbon and C18 the proton acceptor for the proton transfer step following the hydride transfer. For the hydride transfer, the reaction coordinate was defined as $z = r$(CD-H1) - r(C17-H1), where r is the interatomic distance.

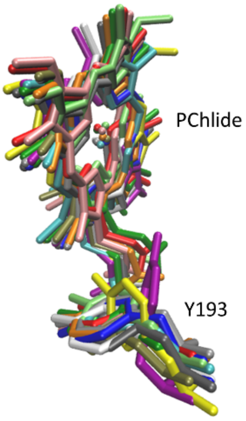

Figure S2. Conformational sampling of Y193 during an unrestrained 100 ns MD simulation of TePOR ternary complex. Representative structures were generated by single linkage clustering of PChlide and the surrounding residues Y193, K197, T145 and C226 with a cut-off of 0.9 Å. Each color represents a different cluster.

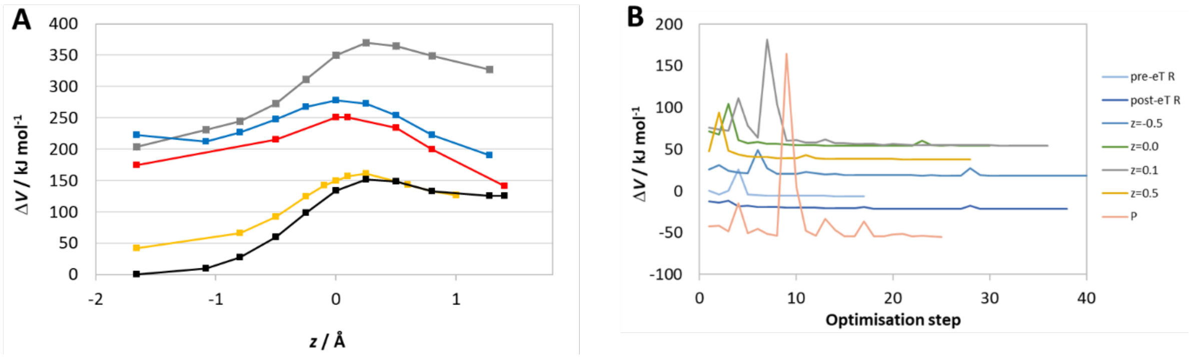

Figure S3. Potential energy barriers for H-transfers, on the following electronic surfaces: the singlet ground state from the pre-eT geometry (black) and post-eT geometry (orange); the singlet excited state, (NDH/PChlide)*, after vertical excitation from the pre-eT geometry (grey); the (NDH^+/$PChlide^-$) state in the pre-eT geometry (blue); the (NDH^+/$PChlide^-$) state after TDDFT energy minimization (red). (B) Convergence of the TDDFT energy minimizations energies, relative to the (NDH/PChlide)* state of the pre-eT geometry. Molecular orbitals were used to confirm that each point along the potential energy surfaces lie on the same electronic surface (Figure S3).

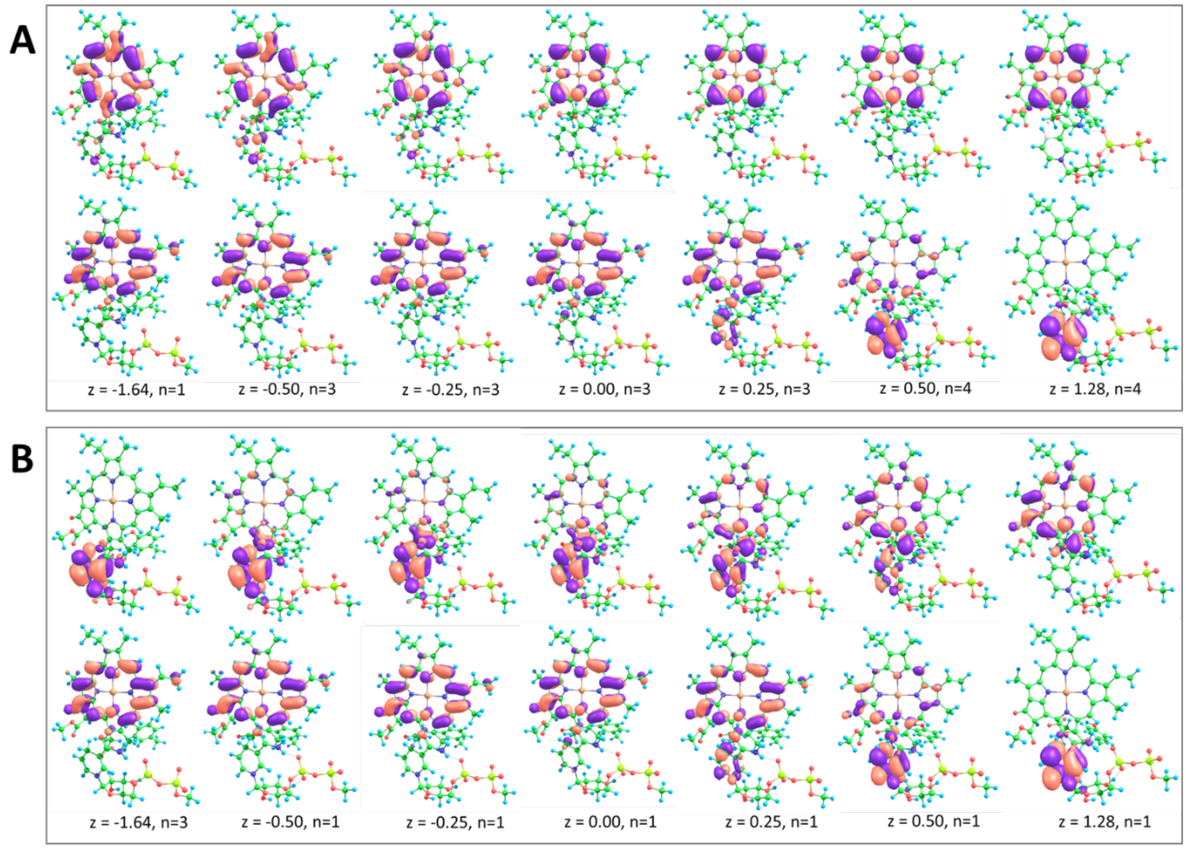

Figure S4. Molecular orbitals that dominate the excited state transitions in TDDFT calculations of selected structures along the H-transfer potential energy scans (Figure S3) for (A) the (NDH/PChlide)* and (B) the (NDH^{+}/$PChlide^{-}$) electronic surfaces in the pre-eT geometry. Top row: donor orbital; bottom row: acceptor orbital; the reaction coordinate (*z*) and transition number (n) are shown below each.

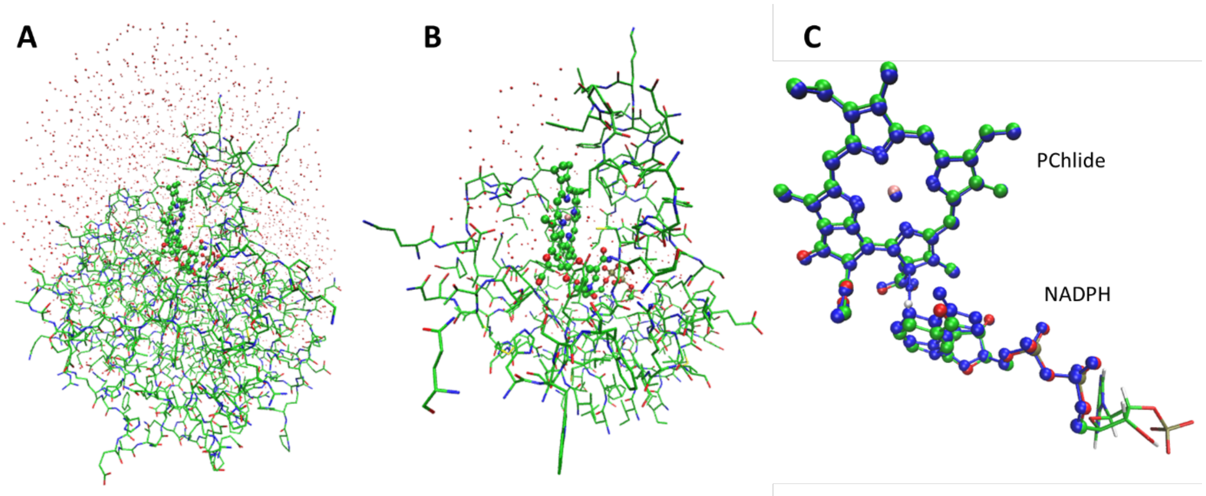

Figure S5. QM/MM model of POR ternary complex: (**A**) entire model, with QM atoms shown using the ball-and-stick representation; (**B**) unrestrained region; (**C**) QM atoms in energy minimized reactant (green) and transition (blue) states in ball-and-stick representation, with the MM part of NADPH from the reactant shown as sticks. Hydrogen atoms omitted for clarity, except the transferring hydrogen atom in **C**.

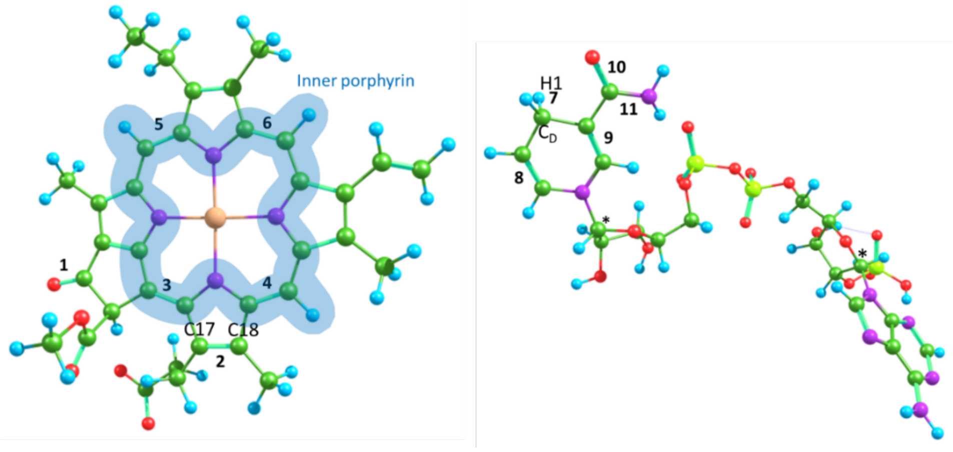

Figure S6. DFT models of PChlide and NADPH. Atoms marked with * were kept fixed during the energy minisation of the NADPH model. C17 is the hydride acceptor, C18 the proton donor for the subsequent step, C_D is the hydride donor and H1 the transferred hydrogen atom. The numbered bonds are those listed in Table S2, and the blue area indicates the inner porphyrin ring as used for charge distributions in Table S1.

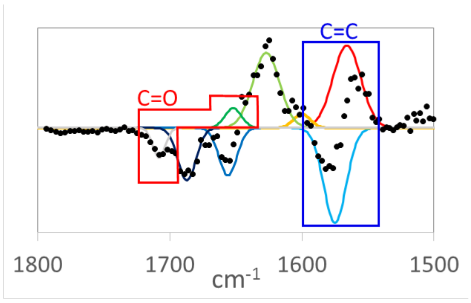

Figure S7. Experimental IR difference spectra (black dots) between “reactive ICT” state and ground state in TePOR fitted to a sum of Gaussian functions (adapted from reference [1]). The peaks in the C=C and C=O regions, with downshifts of ~15 and ~60 cm^{-1}, respectively, are shown by the blue and red boxed regions.

Supplementary Tables

Table S1. Total PChlide charge and charge distribution around the porphyrin for different PChlide electronic states from an isolated PChlide molecule and the PChlide/NDH/Tyr model. The inner and outer porphyrin rings are defined in Figure S5. All values are Natural Charges calculated at the M06-2X/6-31G(d,p) level of theory, but the M06/6-31G(d,p) values are also given in brackets for the PChlide/NDH/Tyr model.

	PChlide			PChlide/NDH/Tyr			
	S0	S1	PChlide$^-$	S0[1]	S1[1]	Orb. swap triplet[2]	S(eT)[3]
Total	-1.00	-1.00	-2.00	-0.98 (-0.98)	-0.98 (-1.00)	-1.83	-1.83 (-1.92)
Inner porhyrin	-1.53	-1.36	-1.74	-1.53 (-1.51)	-1.45 (-1.43)	-1.97	-1.92 (-1.94)
Outer porphyrin	-1.21	-1.38	-1.69	-1.19 (-1.22)	-1.28 (-1.32)	-1.69	-1.63 (-1.72)

[1]The S0 and S1 states were calculated in the pre-eT geometry
[2]The post-eT geometry energy minimised with a spin-multiplicity of 3 after orbital swap
[3]The singlet state corresponding to eT from NDH to PChlide, in the post-eT geometry

Table S2. Bond lengths (in Å) and changes in bond lengths after electron transfer in isolated PChlide / NADPH molecules and the pre-and post-eT optimized PChlide/NDH/Tyr model.

		Isolated PChlide / NADPH			PChlide/NDH/Tyr model		
No.[1]	Bond	*r*(pre-eT)	*r*(post-eT)	Δ*r*	*r*(pre-eT)	*r*(post-eT)	Δ*r*
1	C=O	1.213	1.226	0.013	1.213	1.226	0.013
2	C16-C17	1.366	1.361	-0.005	1.369	1.366	-0.003
3	C=C	1.392	1.386	-0.006	1.392	1.389	-0.003
4	C=C	1.405	1.384	-0.021	1.406	1.386	-0.021
5	C=C	1.394	1.384	-0.010	1.393	1.384	-0.009
6	C=C	1.406	1.386	-0.021	1.406	1.386	-0.020
7	CD-H1	1.100	1.104	0.004	1.102	1.103	0.001
8	C=C	1.334	1.351	0.017	1.335	1.364	0.029
9	C=C	1.353	1.368	0.016	1.352	1.359	0.007
10	C=O	1.236	1.229	-0.007	1.235	1.229	-0.007
11	CO-NH2	1.364	1.340	-0.024	1.362	1.343	-0.019

[1] The corresponding bonds are shown in Figure S5.

Table S3. Excited state energies in kJ mol^{-1} relative to the ground state S0, calculated in both the pre-eT and post-eT geometries.

Geom.	Functional / basis set	S1	S(eT)[1]	T1	T(eT)[1]
pre-eT	M06-2X / 6-31G(d,p)	216.2	305.9	145.4	299.0
	M06-2X / TZVP	211.9	309.7	142.2	272.3
	M06 / 6-31G(d,p)	204.9	224.7	134.6	223.8
	M06-HF / 6-31G(d,p)	210.4	398.8	121.4	304.4
	CAM-B3LYP / 6-31G(d,p)	209.2	322.8	94.1	254.8
	wB97-XD / 6-31G(d,p)	205.9	360.4	90.8	262.5
post-eT	M06-2X / 6-31G(d,p)	246.3	264.0	163.5	262.3
	M06-2X / TZVP	246.0	273.7	164.2	271.6
	M06 / 6-31G(d,p)	184.6 (237.8)[2]	184.6	154.9	188.3
	M06-HF / 6-31G(d,p)	242.8	397.6	138.5	325.3
	CAM-B3LYP / 6-31G(d,p)	246.4	287.0	112.1	270.9
	wB97-XD / 6-31G(d,p)	240.7	318.8	103.8	316.9

[1] The S(eT) and T(eT) states are excited states dominated by transition from a molecular orbital centered on NDH to an orbital centered on PChlide.

[2] For M06 / 6-31G(d,p) in the post-eT geometry, the S(eT) state is the lowest excited state, S1. The second excited state (S2, energy in brackets) corresponds to S1 from the other calculations.

Table S4. Energy (in kJ mol^{-1}) for one-electron reduction of PChlide, one-electron oxidation of NADPH and electron transfer from NADPH to PChlide.

Functional / basis set	$Pchlide^-$ → $Pchlide^-$	NADPH → $NADPH^+$	Pchlide + NADPH –→ $Pchlide^-$ + $NADPH^+$
M06-2X / 6-31G(d,p)	-295.7	476.7	181.0
M06-2X / 6-311+G(d,p)	-321.3	504.0	182.7
M06 / 6-31G(d,p)	-280.5	461.7	181.2
M06-HF / 6-31G(d,p)	-318.2	504.7	186.5
CAM-B3LYP / 6-31G(d,p)	-278.6	455.2	176.6
ωB97-XD / 6-31G(d,p)	-283.4	457.7	174.2

Table S5. Spin-orbit couplings, in cm^{-1}, between of the NDH/PChlide/Tyr model in the pre-eT geometry, calculated from TDDFT calculations using M06/6-31G(d,p) using PySOC.[2]

$\langle S0\|H_{SO}\|T1\rangle$:	0.77	$\langle S2\|H_{SO}\|T1\rangle$:	0.88	$\langle S4\|H_{SO}\|T1\rangle$:	0.07
$\langle S0\|H_{SO}\|T2\rangle$:	0.43	$\langle S2\|H_{SO}\|T2\rangle$:	1.15	$\langle S4\|H_{SO}\|T2\rangle$:	0.07
$\langle S0\|H_{SO}\|T3\rangle$:	0.94	$\langle S2\|H_{SO}\|T3\rangle$:	0.49	$\langle S4\|H_{SO}\|T3\rangle$:	0.10
$\langle S0\|H_{SO}\|T4\rangle$:	0.27	$\langle S2\|H_{SO}\|T4\rangle$:	0.48	$\langle S4\|H_{SO}\|T4\rangle$:	0.10
$\langle S0\|H_{SO}\|T5\rangle$:	0.25	$\langle S2\|H_{SO}\|T5\rangle$:	0.07	$\langle S4\|H_{SO}\|T5\rangle$:	0.78
$\langle S1\|H_{SO}\|T1\rangle$:	1.13	$\langle S3\|H_{SO}\|T1\rangle$:	0.21	$\langle S5\|H_{SO}\|T1\rangle$:	0.59
$\langle S1\|H_{SO}\|T2\rangle$:	0.87	$\langle S3\|H_{SO}\|T2\rangle$:	0.12	$\langle S5\|H_{SO}\|T2\rangle$:	0.40
$\langle S1\|H_{SO}\|T3\rangle$:	0.46	$\langle S3\|H_{SO}\|T3\rangle$:	0.05	$\langle S5\|H_{SO}\|T3\rangle$:	0.12
$\langle S1\|H_{SO}\|T4\rangle$:	0.46	$\langle S3\|H_{SO}\|T4\rangle$:	0.17	$\langle S5\|H_{SO}\|T4\rangle$:	0.29
$\langle S1\|H_{SO}\|T5\rangle$:	0.14	$\langle S3\|H_{SO}\|T5\rangle$:	0.06	$\langle S5\|H_{SO}\|T5\rangle$:	1.59

Table S6. Computed potential energy driving force (ΔV), outer, inner and total reorganisation energies (λ), and the resulting activation energy (in kJ mol^{-1}) for electron transfer from NDH to PChlide and back-electron transfer, in water dichloromethane (DCM) and benzene, after TDDFE energy minimization of each state.

eT					back-eT				
ΔV	λ_{outer}[1]	λ_{inner}[1]	λ	$\Delta V^{\ddagger}$	ΔV	λ_{outer}[2]	λ_{inner}[2]	λ	$\Delta V^{\ddagger}$
-15.4	63.0	31.6	94.7	16.6	-182.1	69.1	30.0	99.1	17.4

[1] For eT λ_{outer} was calculated from the difference between non-equilibrium and equilibrium solvation between (NDH/PChlide)* and (NDH^+/$PChlide^-$) in the post-eT geometry and λ_{innter} was calculated from the difference in energy for the (NDH^+/$PChlide^-$) state in the post-eT and pre-eT geometries.

[2] For back-eT λ_{outer} was calculated from the difference between non-equilibrium and equilibrium solvation between (NDH/PChlide) and (NDH^+/$PChlide^-$) in the pre-eT geometry and λ_{innter} was calculated from the difference in energy for the (NDH/PChlide) state in the post-eT and pre-eT geometries.

Table S7. Vibrational stretching frequencies (in cm^{-1}) for porphyrin ring C=C double bonds, the average frequency for the porphyrin ring C=C double bonds, ⟨C=C⟩, and the carbonyl C=O bond for three electronic states of PChlide: ground state S0, first first excited state S1 and $PChlide^-$. The frequencies have been scaled by a factor of 0.95.

Bond type	Pchlide S0	Pchlide S1	$Pchlide^-$
C=C	1482.3	1469.4	1463.6
	1491.2	1502.7	1477.6
	1517.2	1512.1	1497.5
	1528.5	1537.4	1510.5
	1543.9	1552.0	1516.2
	1567.7	1564.3	1531.2
	1583.0	1577.9	1561.9
	**1597.7**	**1609.7**	**1612.7**
	1602.2	1614.8	1617.0
⟨C=C⟩	1546.0	1548.9	1532.0
C=O	1737.6	1701.6	1662.5

Additional References

1. Heyes, D. J.; Hardman, S. J.; Hedison, T. M.; Hoeven, R.; Greetham, G. M.; Towrie, M.; Scrutton, N. S., Excited-state charge separation in the photochemical mechanism of the light-driven enzyme protochlorophyllide oxidoreductase. *Angew Chem Int Edit* **2015,** *54*, 1512-5.
2. Gao, X.; Bai, S.; Fazzi, D.; Niehaus, T.; Barbatti, M.; Thiel, W., Evaluation of Spin-Orbit Couplings with Linear-Response Time-Dependent Density Functional Methods. *J Chem Theory Comput* **2017,** *13*, 515-524.
